# Unbiased corneal tissue analysis using Gabor-domain optical coherence microscopy and machine learning for automatic segmentation of corneal endothelial cells

**DOI:** 10.1117/1.JBO.25.9.092902

**Published:** 2020-08-07

**Authors:** Cristina Canavesi, Andrea Cogliati, Holly B. Hindman

**Affiliations:** a LighTopTech Corp., West Henrietta, New York, United States; b The Eye Care Center, Canandaigua, New York, United States

**Keywords:** optical coherence tomography, microscopy, machine learning, ophthalmology, corneal imaging, eye banking

## Abstract

**Significance:** An accurate, automated, and unbiased cell counting procedure is needed for tissue selection for corneal transplantation.

**Aim:** To improve accuracy and reduce bias in endothelial cell density (ECD) quantification by combining Gabor-domain optical coherence microscopy (GDOCM) for three-dimensional, wide field-of-view ($1\;{\mathrm{mm}}^{2}$) corneal imaging and machine learning for automatic delineation of endothelial cell boundaries.

**Approach:** Human corneas stored in viewing chambers were imaged over a wide field-of-view with GDOCM without contacting the specimens. Numerical methods were applied to compensate for the natural curvature of the cornea and produce an image of the flattened endothelium. A convolutional neural network (CNN) was trained to automatically delineate the cell boundaries using 180 manually annotated images from six corneas. Ten additional corneas were imaged with GDOCM and compared with specular microscopy (SM) to determine performance of the combined GDOCM and CNN to achieve automated endothelial counts relative to current procedural standards.

**Results:** Cells could be imaged over a larger area with GDOCM than SM, and more cells could be delineated via automatic cell segmentation than via manual methods. ECD obtained from automatic cell segmentation of GDOCM images yielded a correlation of 0.94 (p<0.001) with the manual segmentation on the same images, and correlation of 0.91 (p<0.001) with the corresponding manually counted SM results.

**Conclusions:** Automated endothelial cell counting on GDOCM images with large field of view eliminates selection bias and reduces sampling error, which both affect the gold standard of manual counting on SM images.

## 1 Introduction

Endothelial cells show poor regenerative capacity *in vivo* and are critical for preserving corneal transparency vis-a-vis their fluid pumping mechanism that maintains appropriate hydration status and clarity of the stroma. We are limited in our ability to assess corneal endothelial function, but evaluation of endothelial density is used as a marker for corneal endothelial health.^1^ Quantitative assessments of endothelial cell density (ECD) and morphology with specular microscopy (SM) are performed at eye banks to evaluate donor tissue quality.^2^^,^^3^ To minimize the risk of corneal donor tissue contamination, SM is performed with the donor corneal tissue immersed in a preserving medium (Optisol GS or Life 4°C) within its polymethyl methacrylate (PMMA) storage and viewing chamber (Bausch + Lomb, Krolman, Stephens or Numedis). In order to achieve good-to-excellent imaging quality, it is recommended to warm the tissue at room temperature for at least 2 h for corneas stored in Optisol GS and at least 2.5 h for Life 4°C.^4^ Rapid warming of tissue in an incubator was shown to be a safe alternative to room temperature warming.^5^ SM, which has been used in eye banks since the 1980s, provides a magnified view of the endothelial layer, over a field of view typically of 300  μm×400  μm. Cell counting is performed by manually selecting ∼50 to 100 cells in the field of view and using one of various extrapolation methods (comparison method, frame method, corner method, and center to center method).^6^ Since the cells are selected manually, the analysis can suffer from selection bias and interoperator variability. The Cornea Donor Study Group evaluated 663 SM images and compared the results obtained by individual eye banks and by the Cornea Reading Center; only 65% of the images yielded a difference in cell count under 10%.^7^ Factors such as image quality, death to preservation time, and presence of epithelial defects or of Descemet’s membrane folds, as well as the choice of method used for extrapolating the ECD, all impact the variability in ECD between eye banks.

ECD was adopted as a medical standard by the Eye Bank Association of America (EBAA) in 2001. The EBAA guidelines do not provide a strict cutoff based on ECD for selection of corneal tissue for transplant and leave the decision to the discretion of eye bank medical directors. Most eye banks indicate 2000 as the minimum ECD requirement for penetrating keratoplasty; however, surgeons commonly request and prefer higher ECDs (e.g., above 2300 to $2500\;{\text{cells}/\mathrm{mm}}^{2}$).^3^ In addition, 6-month postoperative ECD can be an important metric to predict endothelial graft failure after penetrating keratoplasty.^8^

Several algorithms for the automatic segmentation of the endothelial cells, i.e., identification of the individual cells, from images acquired with SM or confocal microscopy have been proposed over the past three decades. Some of the early approaches, some of which were included in commercial implementations,^9^^–^^11^ relied on thresholding cell edges and on morphological analysis of the shape of the cells after noise filtering and contrast enhancement.^12^^–^^14^ These methods lack sufficient robustness to deal with images of varying quality, as they are prone to under- and oversegmentation in regions with poor image quality. The authors did not provide any quantitative measure of image quality. Another set of methods used image characteristics, such as the hexagonality of the endothelial cells, as a filtering feature.^15^^,^^16^ While being more robust than earlier approaches, the effectiveness of these methods is hampered by their inability to determine an optimal threshold level for the images. Another set of methods rely on watershed segmentation.^17^^–^^20^ However, this approach is generally susceptible to under- or oversegmentation, especially in areas with poor image contrast.

Machine learning approaches have also been proposed. Scarpa and Ruggeri developed a genetic algorithm to fit pixel intensities with hexagonal meshes.^21^ More recently, neural networks have been applied successfully to automatically segment SM images. One advantage of neural networks is that they do not require prespecified features. Fabijańska^22^ applied a convolutional neural network (CNN) based on the U-Net to a dataset of 30 SM manually annotated images of good quality and achieved an area under the ROC curve of 0.92. Daniel et al.^23^ applied the U-Net architecture to a dataset of 385 manually annotated SM images and demonstrated an $R^{2}$ from Pearson’s correlation between automated and manually annotated cell counting of 0.96, while a traditional approach using grayscale morphology and water shedding correlated with the ground truth with $R^{2}$ as low as 0.35. Joseph et al. also use U-net in their proposed end-to-end system for semiautomatic cell segmentation of SM endothelial images. The proposed processing pipeline includes image flattening, U-net deep learning, and user-assisted postprocessing. The system shows human-comparable performance, i.e., 92% of cells from the test set were scored as acceptably segmented by a human evaluator, with a full annotation being able to be completed in under 5 min per image.^24^ Auksorius et al.^25^ also recently proposed a similar pipeline for volumetric images obtained with Fourier-domain full-field optical coherence tomography (OCT). The proposed pipeline used a modified U-Net neural network for the segmentation, trained with two OCT images and four SM images.

Gabor-domain optical coherence microscopy (GDOCM) is a high lateral resolution variant of Fourier-domain OCT.^26^^,^^27^ GDOCM incorporates concepts of OCT (imaging depth of 2 to 3 mm) and confocal microscopy (micron-scale lateral resolution). GDOCM breaks the depth-invariant cellular resolution limit of OCT by incorporating a liquid lens in a custom microscope with 0.18 numerical aperture (corresponding to a lateral resolution of 2  μm), which is used to dynamically refocus the beam at different depths.^28^ A microelectromechanical systems (MEMS) mirror is used to steer the beam over a field of view of 1  mm×1  mm.^29^ Multiple volumetric images are acquired and fused together. GDOCM was previously used with contact imaging modality to produce 3D images of corneal tissue with isotropic resolution of 2  μm.^30^^–^^34^ Manual counting of endothelial cells from 180 GDOCM images (six repeated images at five different locations on six corneas) yielded repeatability under 2.3% for six repeated images at each location.^32^

The capability to image the cornea in 3D over its full thickness with micrometer-scale resolution allows for accurate measurement of thickness^35^ and evaluation of stromal opacities.^36^ This can be applied to investigating corneas affected by microbes (acanthamoeba and fungal elements),^37^ corneal nerves,^34^ corneal dystrophies (epithelial, stromal, and endothelial),^31^ scars, incisions, and Descemet’s membrane detachments. In eye banking, it can be useful for pre- and postprocessing comparisons.^38^ In addition to the 3D imaging capability with cellular resolution, GDOCM has the major advantage of a large field of view of $1\times 1\;{\mathrm{mm}}^{2}$, which is 4 to 10 times larger than the capability of commercial SM commonly used at eye banks, as illustrated in Table 1.

**Table 1:** Comparison of commercial SM for eye bank corneal endothelium analysis and the GDOCM instrument.

Company	HAI Laboratories	Konan	Topcon	LighTopTech
Model	EB-3000xyz	CellChek D+	SP-1P	GDOCM 4D™
Technology	SM	SM	SM	GDOCM
Analysis area	$0.225\;{\mathrm{mm}}^{2}$ (450 μm×500 μm)	$0.12\;{\mathrm{mm}}^{2}$ (400 μm×300 μm)	$0.1375\;{\mathrm{mm}}^{2}$ (250 μm×550 μm)	$1\;{\mathrm{mm}}^{2}$ (1 mm×1 mm)

In this paper, we report on the first demonstration of noncontact GDOCM to noninvasively image corneal tissue stored in viewing chambers, and on the development of numerical methods to digitally flatten the endothelium and extract quantitative assessment of ECD from GDOCM images of corneal tissue. The proposed methods are validated against the gold standard of manual cell counting from SM images.

## 2 Methods

### 2.1 Noncontact, 3D Large Field-of-View Corneal Imaging with Gabor-Domain Optical Coherence Microscopy

**Fig. 1:**
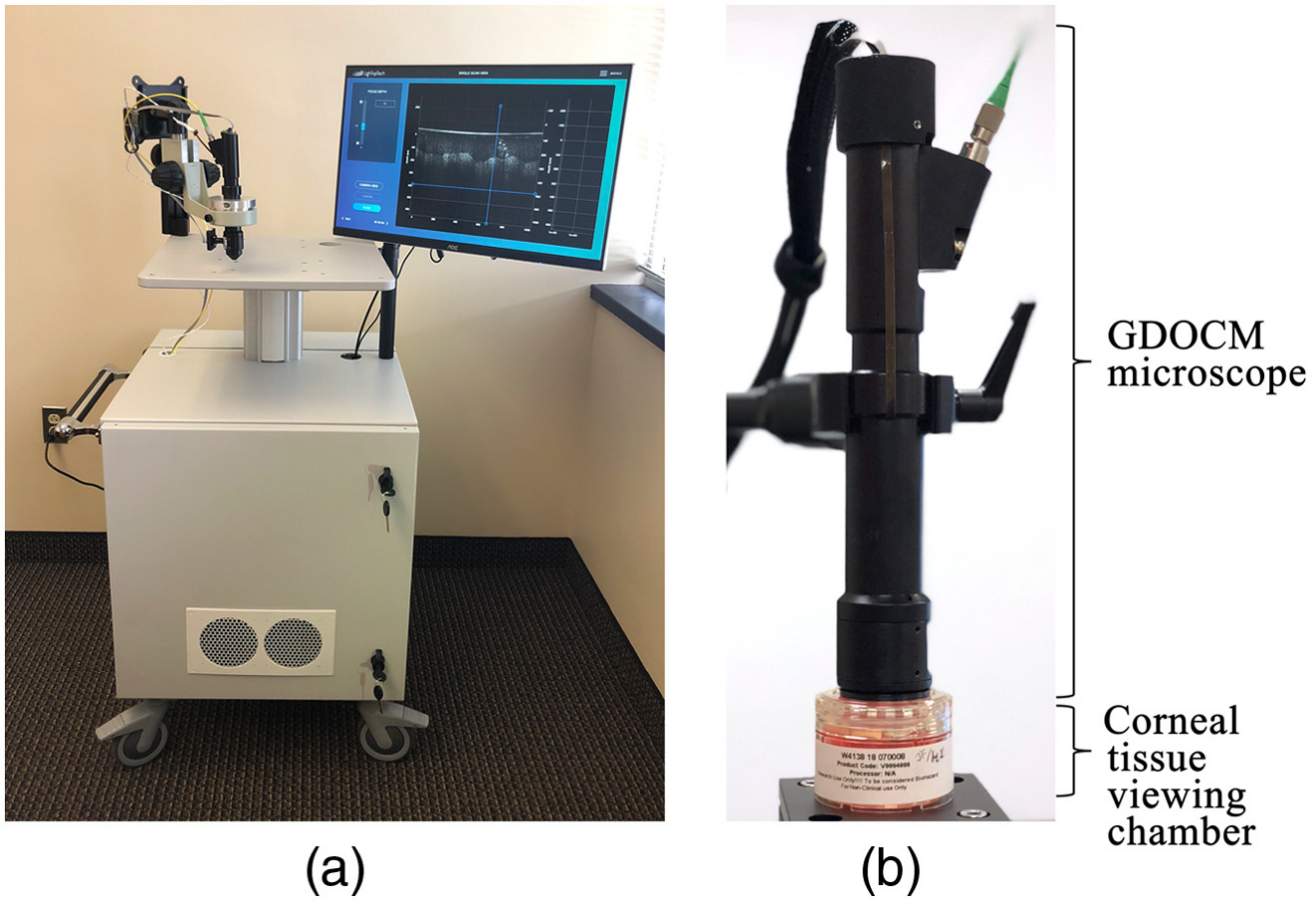
(a) GDOCM system and (b) GDOCM microscope operating at 15-mm working distance to image corneal tissue through a viewing chamber. There is an air gap between the microscope and the viewing chamber.

GDOCM was first developed for contact imaging, which is preferred in applications that benefit from index-matching between the microscope and the sample, such as dermatology.^27^^,^^39^^,^^40^ The contact imaging modality, however, is not desirable for corneal imaging, since physical contact may cause damage to the tissue, and requires exposing the tissue to the outside environment. The GDOCM microscope was recently redesigned to support two imaging modalities: contact and noncontact with 15-mm working distance. The GDOCM system shown in Fig. 1(a) can be used to image corneal tissue stored in viewing chambers using a noncontact imaging modality, as shown in Fig. 1(b). For corneal tissue imaging with both GDOCM and SM, the PMMA viewing chamber causes losses in the signal acquired due to the specular reflection at the air/PMMA interface, and due to the scattering and absorption inside the material; in the case of GDOCM, high signal-to-noise ratio images of the corneal tissue can still be achieved due to the capability to dynamically focus the beam on the tissue below the lid of the container.

Data from six corneas previously imaged with GDOCM with contact modality (180 images total) and manually counted^32^ were used to train the CNN for the automatic segmentation of endothelial cells. Five locations on each of six corneas were imaged; six repeated imaged were taken at the same location after repositioning the sample under the microscope, for a total of 180 images. The purpose of repeating the imaging at the same location after repositioning the sample under the microscope was to augment the dataset while having a limited number of corneas available, and to acquire multiple images affected by real imaging issues, such as noise and other imaging artifacts potentially introduced by the operator when positioning the probe.

When introducing a new imaging modality, such as GDOCM, validation against the gold standard (SM in this case) is necessary to demonstrate noninferiority of the new solution. To assess the performance of the automated cell counting procedure, 10 donor corneas were imaged with SM and with GDOCM in noncontact modality through a viewing chamber (endothelium side up). Optisol GS (Bausch + Lomb, Rochester, New York) was used as the storage medium. The GDOCM imaging configuration is shown in Fig. 1(b). The donor tissue was warmed at room temperature for 2 h prior to imaging.

**Fig. 2:**
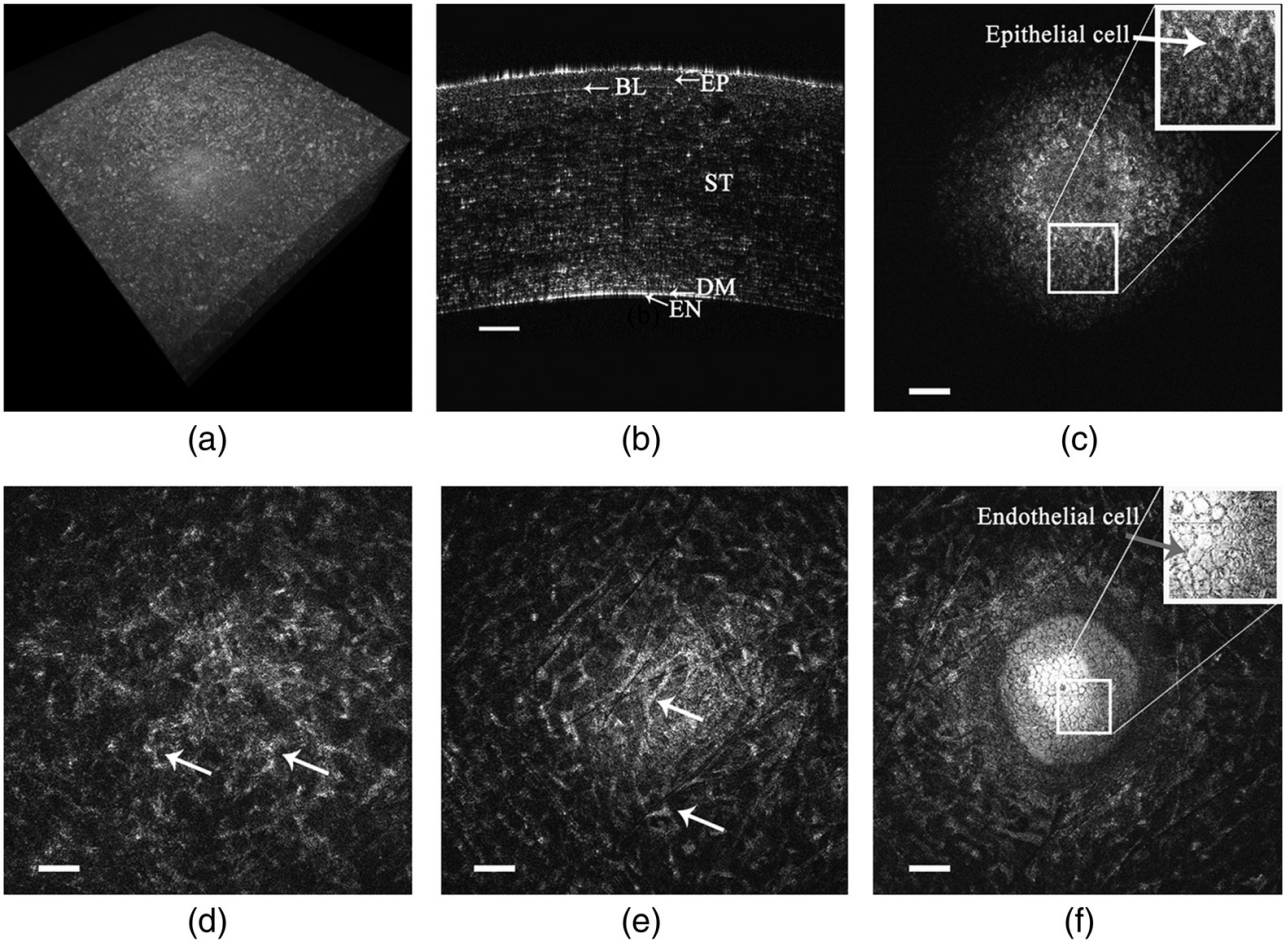
GDOCM images of an *ex vivo* human cornea (field of view $1\;{\mathrm{mm}}^{2}$) imaged through the viewing chamber. (a) 3D view. (b) Full-depth cross-sectional image showing the corneal layers: EP, epithelium; BL, Bowman’s layer; ST, stroma; DM, Descemet’s membrane; EN, endothelium. (c) *En face* view of the epithelium. *En face* images of the (d) middle and (e) posterior stroma reveal stromal keratocytes (white arrows). (f) *En face* view of the transition between stroma and endothelium, with endothelial cells visible. The bars are 100  μm.

An example of the 3D high-definition, wide field-of-view imaging capability of GDOCM is shown in Fig. 2.

**Fig. 3:**
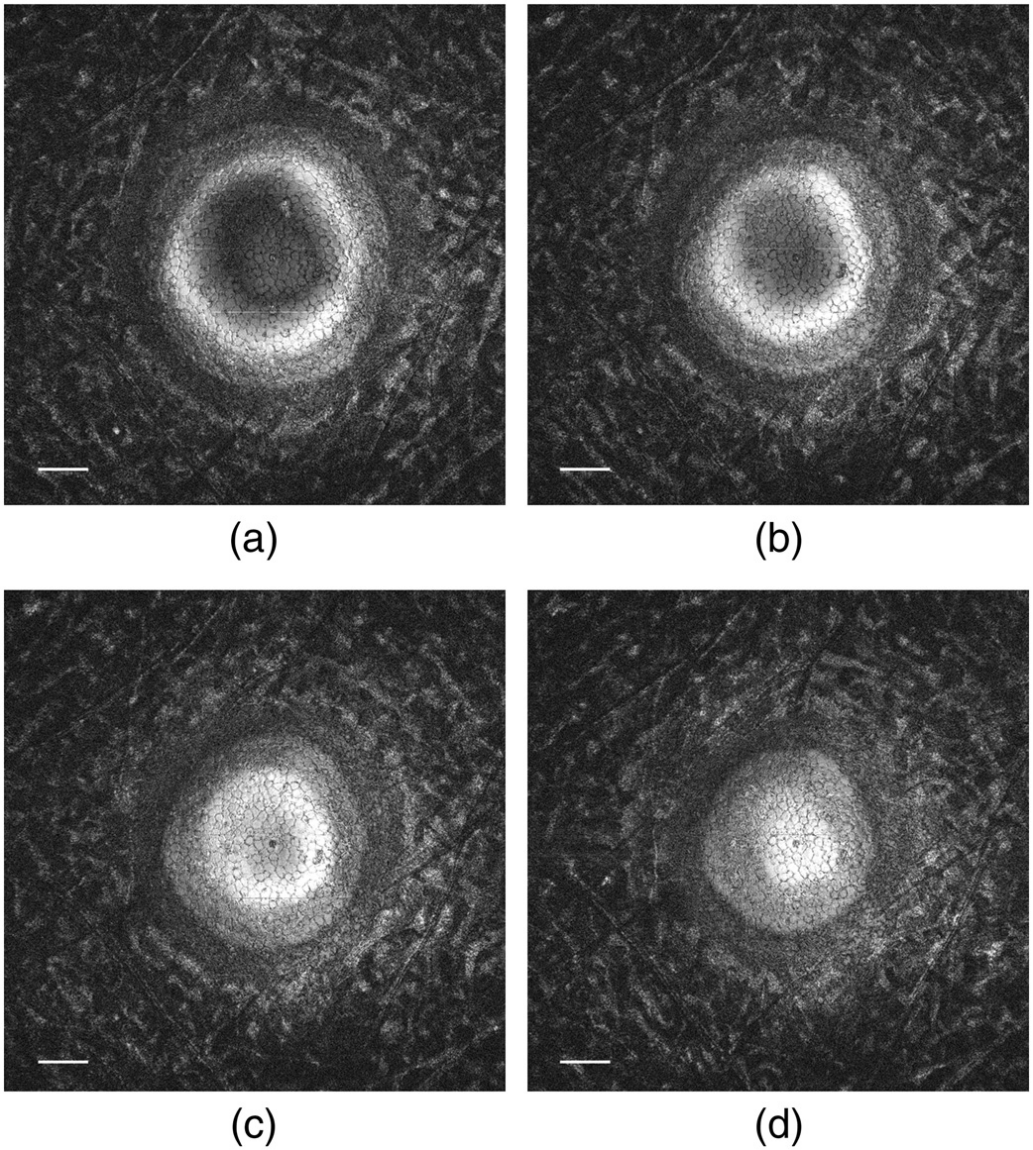
*En face* views at the transition between stroma and endothelium. (a)–(d) Separated in depth by 3  μm each. Bars: 100  μm. The hyper-reflective endothelial cells appear brightest when in focus.

The 3D corneal images acquired by GDOCM, such as SM images, are affected by the curvature of the endothelium. As a consequence, it is not possible to visualize the endothelial cell layer in a single *en face* image, as shown by Fig. 3.

Each GDOCM *en face* view is separated by ∼1.5  μm, which corresponds to the axial sectioning capability of the system. The *en face* images shown in Figs. 3(a)–3(d) span a total depth of 9  μm and are each separated by ∼3  μm; it can be seen that only a fraction of the total endothelial cells is in focus and clearly visible in each image. The hyper-reflective endothelial cells appear the brightest when they are in focus. In addition, cells at the center of the cornea are brighter than at the periphery, since there is some loss of light at the periphery due to the natural curvature of the cornea causing some of the backscattered light not to be collected by the microscope. As such, the effective area considered in the analysis was $0.2\;{\mathrm{mm}}^{2}$, corresponding to the central portion of the cornea with a diameter of 0.5 mm. It should be noted that this constitutes a significant improvement (6 to 12×) in the number of cells counted over the current practice, which consists of eye bank technicians manually counting only a total of 50 to 100 cells within the $0.12\;{\mathrm{mm}}^{2}$ area imaged with SM. This improvement is due to two concurrent factors: (1) the area of analysis is increased with GDOCM compared to the state-of-the art SM instruments, and (2) with automated cell counting all cells in the area of analysis are assessed, while the manual counting performed by eye bank technicians is done by assessing only 50 to 100 cells. With a typical ECD of $3000\;{\text{cells}/\mathrm{mm}}^{2}$, the number of cells present in a $0.2\text{-}{\mathrm{mm}}^{2}$ area is 600, which is 6 to 12 times the number of cells manually assessed (50 to 100).

### 2.2 Automatic Segmentation and Numerical Flattening of the Endothelium

**Fig. 4:**
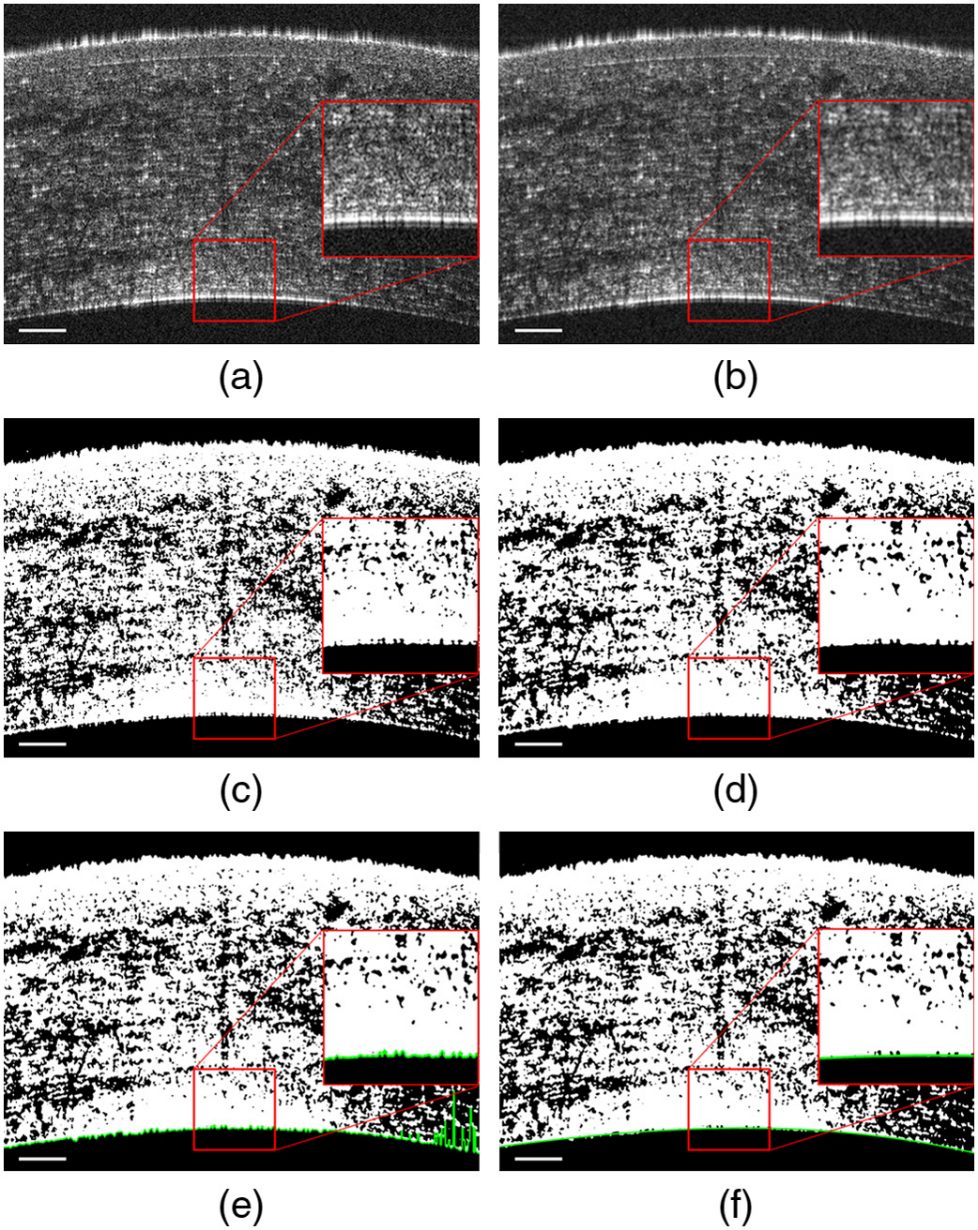
Automated segmentation of the endothelium from a GDOCM image shown here in a 2D cross-section. (a) Original image. (b) After Gaussian blur (step 1). (c) After Huang binarization (step 2). (d) After median filtering (step 3). (e), (f) Segmented endothelial surface in green (e) before and (f) after RANSAC fitting (step 4). The bars are 100  μm.

Numerical methods can be implemented to digitally flatten the endothelium in a 3D GDOCM image. Previous flattening attempts of GDOCM images consisted of detecting the peak in each A-scan (1D depth profile) and repeating this step to the entire volumetric image to extract the endothelial surface; after polynomial fitting, the pixels of the 3D image were shifted to produce a flattened view of the endothelium.^31^^,^^32^ The drawback of this approach is that artifacts are often present in the flattened image due to the 1D approach of this method. Here, we propose the 3D approach illustrated in Fig. 4, which consists of the following steps. Step 1: Gaussian blur for noise reduction. Gaussian blur, a form of low-pass filter, is a standard noise reduction technique used in image processing to reduce the sharpness images, thus reducing noise artifacts from the acquired image. Step 2: Segmentation of the cornea using Huang binarization.^41^ Ideally, the acquired volume should be bimodal, i.e., have bright voxels over the entire cornea and dark voxels elsewhere. In practice, the volume is still noisy, even after denoising. The Huang binarization attempts to find the optimal binarization threshold by minimizing the fuzziness of the image. Step 3: Median filtering to reduce binarization artifacts. Binarization can increase the noise in the image, especially the so-called salt-and-pepper noise. Median filtering is a well-established image processing technique to reduce this type of noise.^42^ Step 4: Identification of the endothelial surface with RANSAC fitting.^43^ Step 5: Max-intensity projection of a slice below the fitted surface to account for estimation errors in steps 2 and 3. The outcome of the 3D flattening approach is an artifact-free *en face* view of the endothelium.

### 2.3 Automated Cell Segmentation and Counting with Machine Learning

**Fig. 5:**
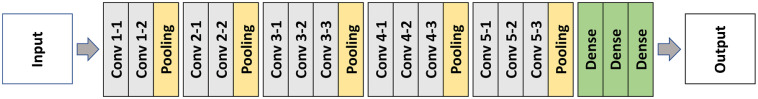
VGG-16 architecture^44^ used for automated endothelial cell segmentation. VGG-16 is a type of CNN. CNNs, also called shift invariant artificial neural networks, are a type of artificial neural network` specially designed for image processing and modeled after the visual cortex of animals. The convolutional layers (denoted by the prefix Conv in the figure) mimic the functions of visual receptors, while the pooling layers filter the most salient activations from the lower layers. Dense layers are regular fully connected multilayer perceptrons, used for classification.

A dataset with 180 GDOCM images (obtained from imaging six corneas at five locations each), and the corresponding manual tracing of the endothelial cells over the central 0.5-mm diameter of the flattened endothelium was used for training and testing a CNN–VGG-16 (see Fig. 5).^44^ We applied a transfer learning approach using the pretrained VGG-16 network. The network is pretrained on more than a million images from the ImageNet database. The pretrained model has already learned to extract features from different image categories and can improve the classification results even with small training datasets. Data augmentation was also used by applying randomly distributed translations of ±10  pixels in both directions, specular reflections and rotations. Scaling and skewing were not applied to preserve the morphology and the sizes of the cells. We trained the CNN six times using a leave-one-out cross-validation strategy.^45^ The training was done on five manually traced corneas (150 images) and tested on the sixth cornea (30 images); this procedure was repeated six times, leaving out a different cornea for testing each time.

Each pixel of the images in the dataset was annotated as belonging to one out of three classes: cell border, cell interior, and noise. For the training, we used stochastic gradient descent with a momentum of 0.9, an initial learning rate of 1e-3, an L2 regularization of 5e-4, a minibatch size of 16 images, and a maximum of 400 epochs.

**Fig. 6:**
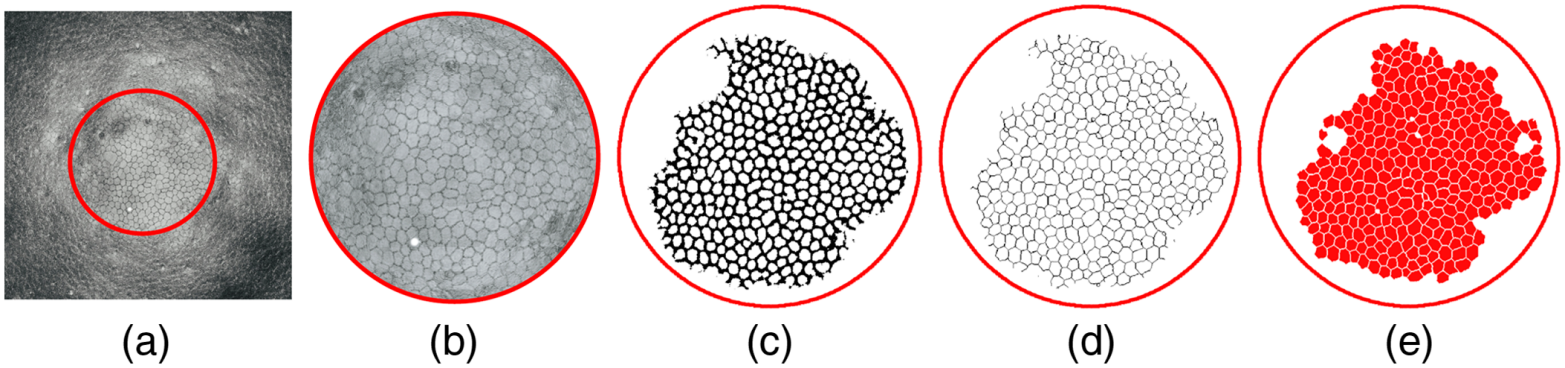
Automated cell counting procedure. (a) After automatic segmentation of the endothelium and numerical flattening, (b) the region with 0.5-mm diameter in the center of the cornea is selected for analysis and cropped, to match the manually annotated data available. (c) The CNN provides automatic segmentation from the image. (d) After skeletonization of the segmented borders, (e) the cell areas can be quantified.

To obtain the segmentation of the cells in the testing step, the raw classification results from the trained network were postprocessed as follows: a binary mask was extracted from the pixels classified as cell border. A two-pixel dilation, to close small gaps in the borders, followed by skeletonization, was applied to the binary mask. Regions smaller than 100 square pixels or larger than 750 square pixels were excluded from the segmentation as representing oversegmentation or noisy areas; the chosen interval included 95% of the cells in the ground truth. An example of the fully automated procedure for ECD analysis from the *en face* view of the flattened endothelium is shown in Fig. 6. The analysis was performed on the central cornea with 0.5-mm diameter to match the manually annotated data available.

### 2.4 Comparison with Specular Microscopy

For comparison with the gold standard of SM, 10 corneas were analyzed at the eye bank using a Konan CellChek D+ SM with the standard evaluation process, in which a technician manually selects 50 to 100 cells and the ECD is obtained via a center/flex center method. The imaging with GDOCM was conducted on the corneas stored in the same viewing chambers as for SM evaluation, and on the same central area of the cornea. After numerical flattening of the endothelium, a CNN was used to automatically obtain the ECD.

## 3 Results

### 3.1 Endothelium Segmentation and Flattening

**Fig. 7:**
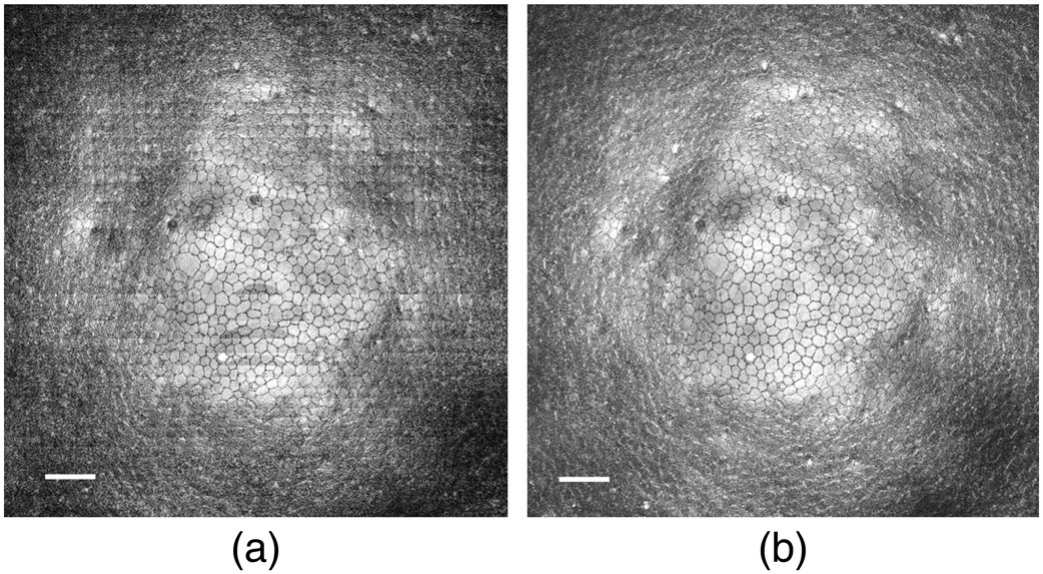
*En face* view of the flattened endothelium. (a) A 1D approach introduces artifacts that appear as lines creating discontinuities in the image, while the proposed 3D approach results in (b) artifact-free flattening.

The automated 3D endothelium segmentation and flattening procedure was applied to 3D GDOCM images to produce *en face* views of the endothelium. A comparison with the 1D approach is shown in Fig. 7.

**Fig. 8:**
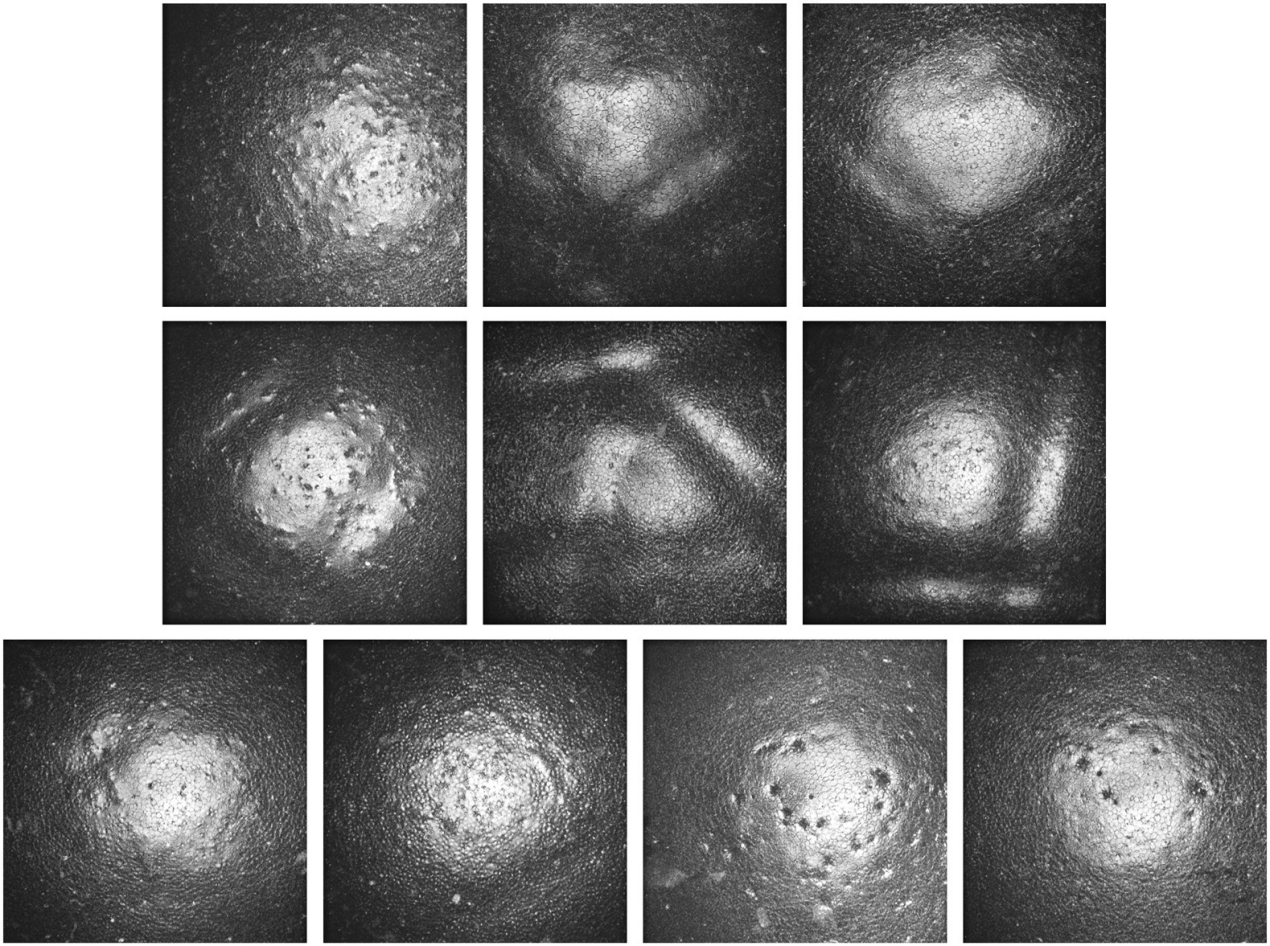
Flattened endothelium for 10 corneas imaged with GDOCM using the noncontact imaging configuration of Fig. 1(b). Dark bands caused by folding of the tissue are visible in a few images.

While the 1D approach can result in artifacts [as shown in Fig. 7(a)] due to mismatch between adjacent points, the 3D approach results in an artifact-free *en face* view of the endothelium [see Fig. 7(b)]. The flattened endothelia for the 10 corneas imaged with noncontact imaging modality through the viewing chambers are shown in Fig. 8.

The darker periphery in the images is caused by the lower scattering signal being collected from regions where the tissue is less normal to the microscope than the center of the cornea.

### 3.2 Automated Cell Segmentation

**Fig. 9:**
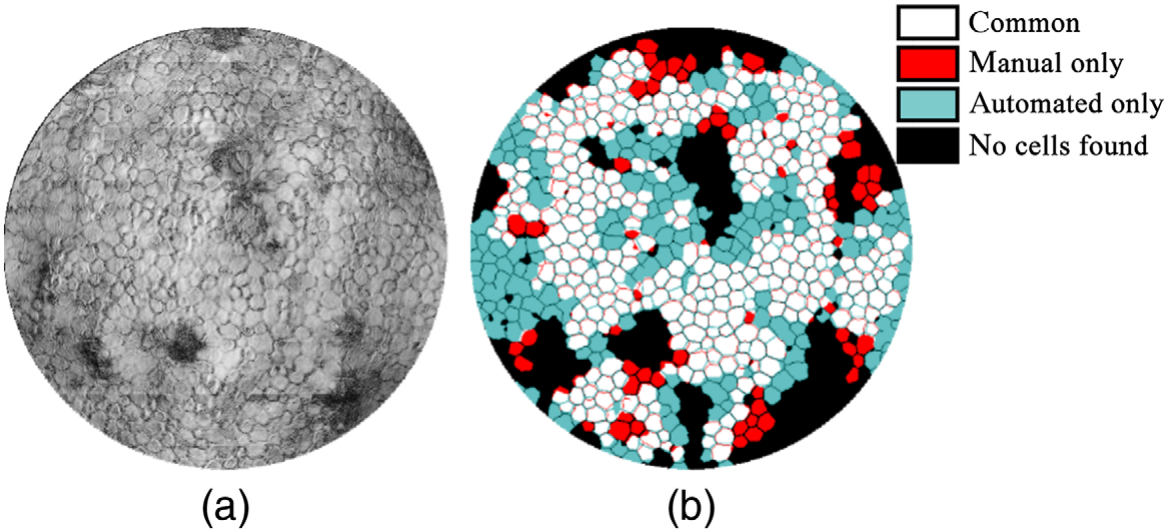
Cell counting results over a region at the center of the cornea with 0.5-mm diameter. The cells colored in white were found by both manual and automated procedures; the cells in teal were found only by the automated procedure; and the cells in red were only found by the manual procedure. In some cases, the automated procedure identified ∼30% more cells than the manual annotation.

The manually annotated dataset available consisted of 180 images from six corneal samples imaged with GDOCM.^32^ The CNN was trained using manually traced images from five corneal samples, for a total of 150 images (six repeated images at five different locations on each cornea). Postprocessing of the identified borders yielded the cell segmentation. The performance of the CNN was tested on 30 images from the remaining cornea (six repeated images at five different locations). This procedure was repeated six times in a leave-one-out cross-validation strategy. An example of the results obtained is shown in Fig. 9 overlaid with the manual annotation.

**Fig. 10:**
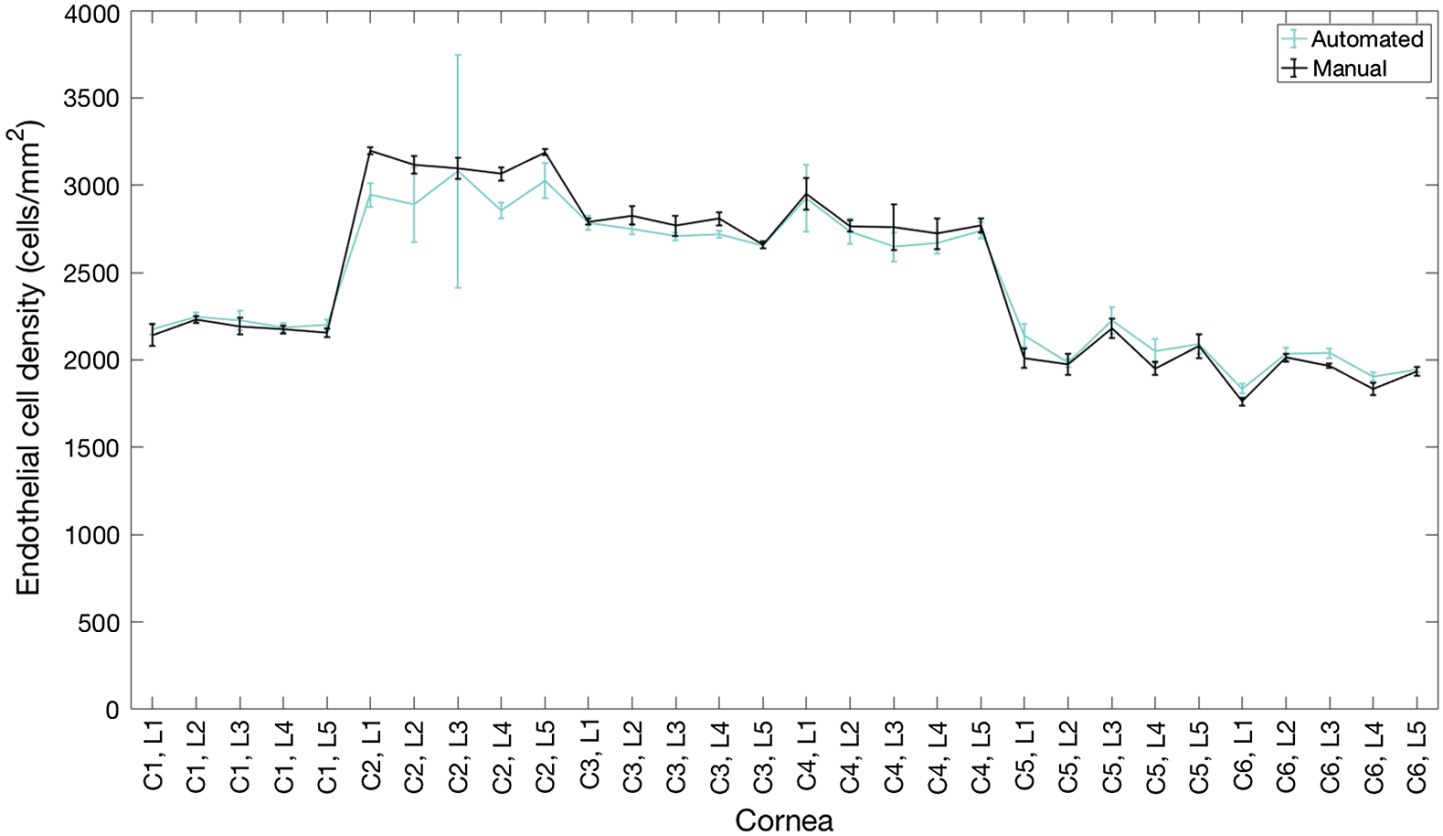
Comparison of cell counting results for manual (black line) and automated (blue line) procedures on six corneas (cornea 1 to cornea 6) imaged at five locations each (L1-L5), with six repeated images at each location (total 180 images). The automated cell counting results were obtained with a leave-one-out cross validation. Error bars indicate the standard deviation for the six repeated measurements at each location.

An average of 316 cells per image was counted from the GDOCM images with the automated procedure, compared to 307 with the manual procedure. The comparison between manual and automated cell counting on cell density values for the corneas used for assessing the CNN performance is shown in Fig. 10. Only cells identified by both methods have been included in this analysis. We chose to only include cells found by both methods, because in reviewing the ground truth we noticed that several instances of cells found by the CNN that had incorrectly not been annotated in the manual analysis. On average, the CNN found 12% more cells than what was in the manual annotation.

**Fig. 11:**
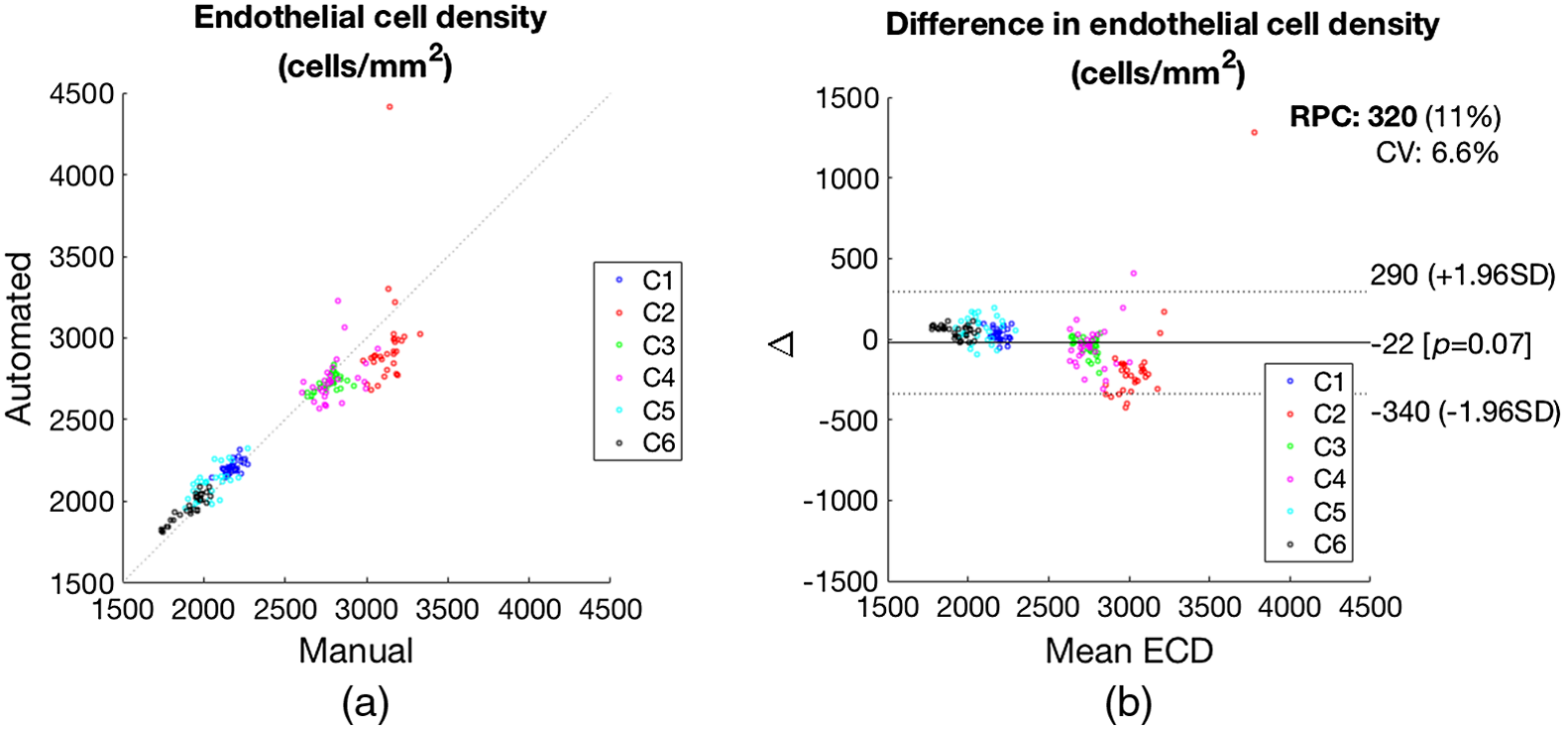
(a) Comparison of manual and automated ECD estimation from GDOCM images for six corneas (C1 to C6), each imaged at five locations with six repetitions (30 images per cornea). (b) Bland–Altman plot showing show the difference between ECD measurements by the automated and manual methods plotted against the mean ECD.

The average difference in ECD between the two modalities was 0.3%, and their Pearson correlation had an $R^{2}$ of 0.94 (p<0.001), as shown in Fig. 11(a).

The difference in mean ECD between the two automated and manual cell count was $22\text{ cells}/{\mathrm{mm}}^{2}$, and Bland–Altman analysis revealed that 95% of the differences in ECD were within $\pm 185\text{ cells}/{\mathrm{mm}}^{2}$ of the mean difference, as shown in Fig. 11(b). The Bland Altman 95% confidence interval includes zero.

**Fig. 12:**
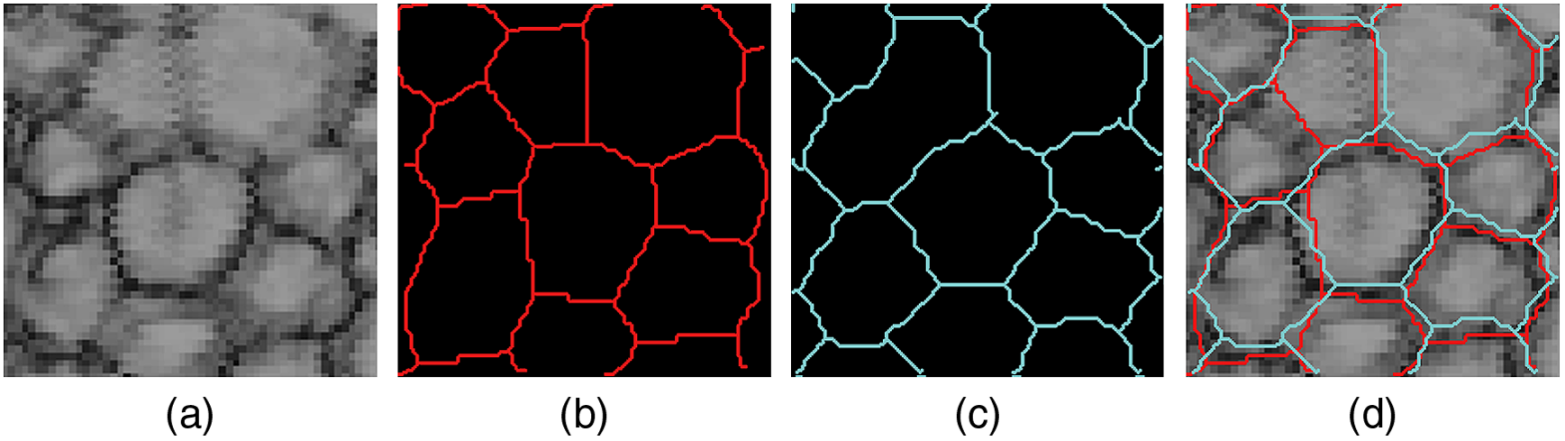
Example of undersegmentation with the automated cell counting method. (a) GDOCM image of the endothelial cells. (b) Manual annotation of the cell borders. (c) Outcome of the CNN, with two cells incorrectly identified as a single cell. (d) Overlay of (a)–(c).

In some cases, the automated procedure resulted in undersegmentation (see Fig. 12), thus causing lower ECD results.

The average Dice score^46^ was 0.66 when computed on all cells, and 0.77 when computed only on ground truth cells (thus excluding false positives due to incomplete ground truth).

### 3.3 Comparison with Specular Microscopy

To assess the GDOCM automatic cell counting relative to SM, 10 additional corneas were imaged for which we had a complete report from the eye bank, including ECD assessed on a Konan CellChek D+ SM. The tissue data are summarized in Table 2. The time between death and tissue preservation was on average 11.1 h (range: 4.5 to 18.6). The GDOCM imaging took place an average of 5.6 days after the SM imaging (range: 2.1 to 7.2).

**Table 2:** Donor age, time between death and cooling, time between death and preservation, time between preservation and SM imaging, and time between SM imaging and GDOCM imaging for 10 corneas imaged in this study.

Cornea #	Age	Time between death and cooling (h)	Time between death and preservation (h)	Time between preservation and SM imaging (h)	Time between SM and GDOCM imaging (h)
1	69	1.3	8.4	9.9	144.5
2	69	1.3	8.3	9.8	167.6
3	69	2.9	18.6	20.3	168.6
4	69	2.9	18.5	20.5	166.7
5	57	1.4	10.3	12.9	173.5
6	57	1.4	10.2	13.0	172.7
7	62	6.5	13.7	9.6	124.3
8	62	6.5	13.8	9.4	124.5
9	66	2.5	4.6	56.7	51.1
10	66	2.5	4.5	56.7	53.2
Average		2.9	11.1	21.9	134.7

**Fig. 13:**
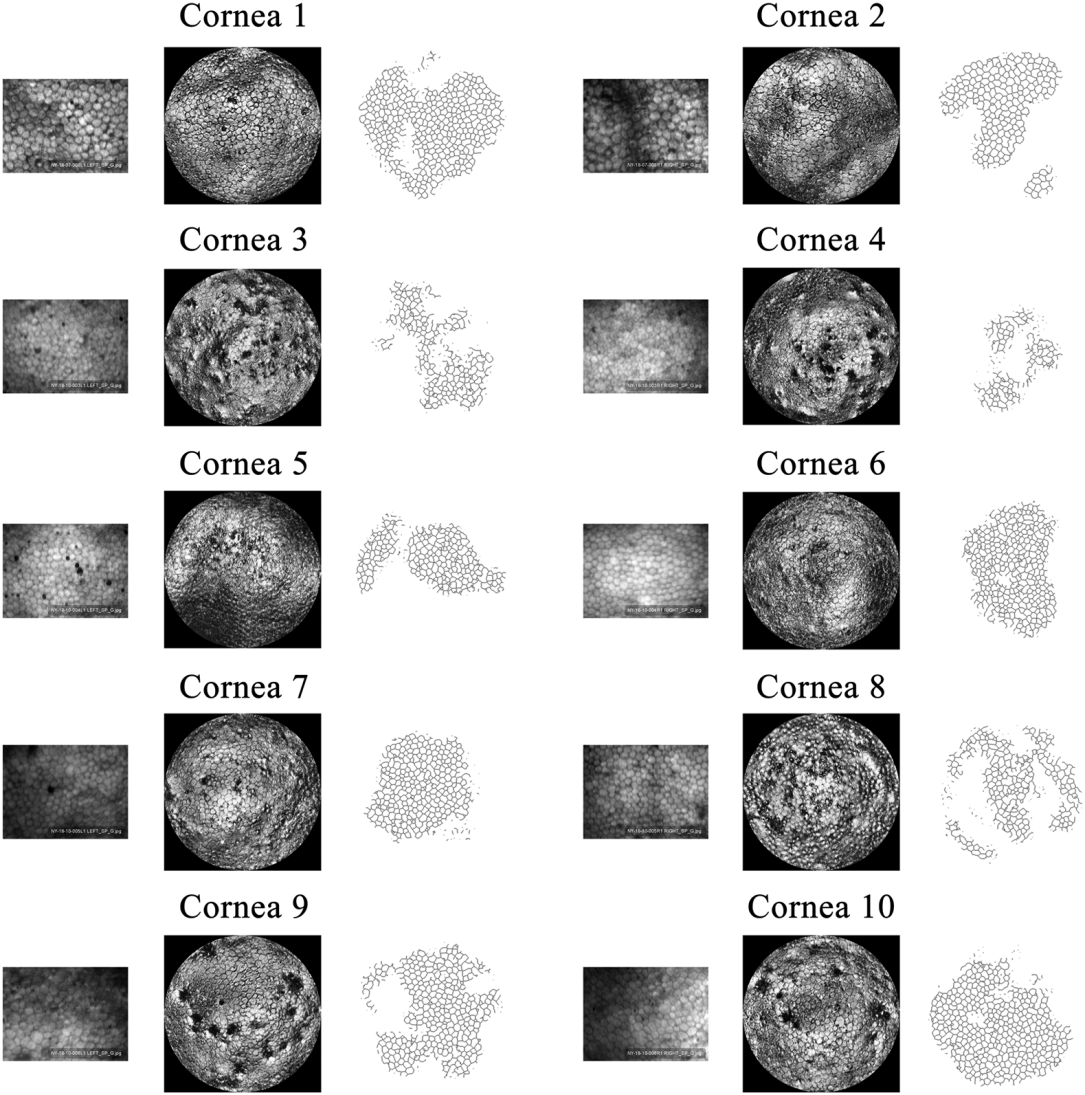
Comparison of (left) SM images, (middle) GDOCM central 0.5-mm diameter images, and (right) automatic segmentation for the 10 corneas of Table 2. Images have the same scale.

The GDOCM automated cell count results were conducted on the central 0.5 mm of each image. The results are shown in Fig. 13.

**Fig. 14:**
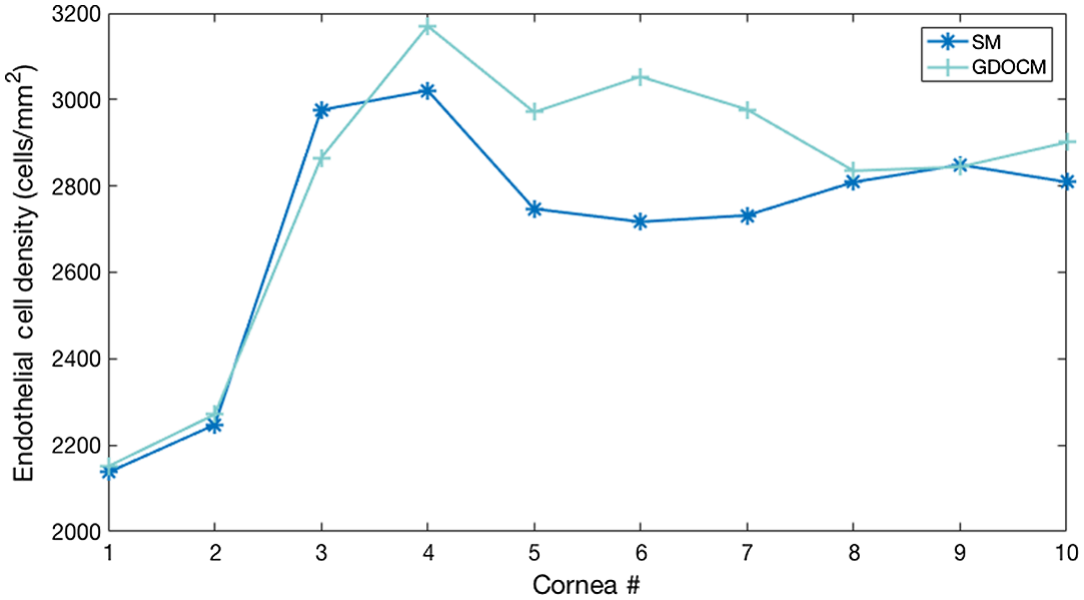
Comparison of semiautomated ECD estimation from SM images and automated ECD estimation from GDOCM images for 10 corneas of Table 2.

The automated cell count results were manually corrected for the cases in which two or more cells were combined in one. An average of 180 cells were counted from the GDOCM images with the automated procedure. The regions imaged with the two modalities were near the center of the cornea but not identical. Also, it should be noted that the SM results likely suffer from selection bias (i.e., the operator only selects a subset of 50 to 100 cells within the field of view). These factors can account for differences in the counts. The results are shown in Table 3 and Fig. 14.

**Table 3:** Cell counting from SM images (semiautomated with manual cell selection) and GDOCM images (automated) for the ten corneas imaged in this study.

Cornea #	SM ECD ($\text{cells}/{\mathrm{mm}}^{2}$)	GDOCM ECD ($\text{cells}/{\mathrm{mm}}^{2}$)	Percent difference in ECD (%)
1	2137	2151	0.7
2	2247	2272	1.1
3	2976	2865	−3.7
4	3021	3171	5.0
5	2747	2971	8.2
6	2717	3053	12.4
7	2732	2977	9.0
8	2809	2835	0.9
9	2849	2844	−0.2
10	2809	2901	3.3

**Fig. 15:**
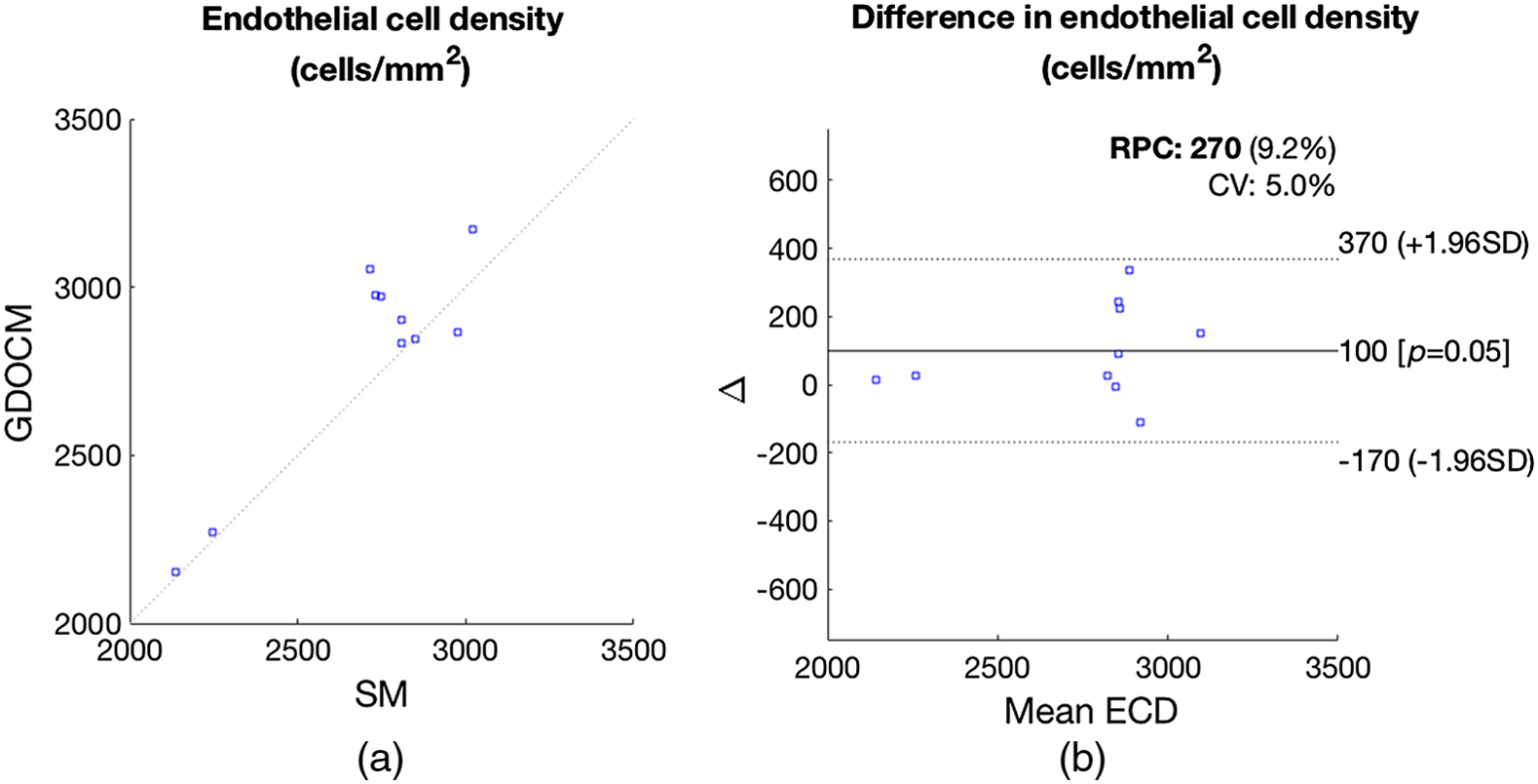
(a) Comparison of semiautomated ECD estimation from SM images and automated ECD estimation from GDOCM images for the 10 corneas of Table 2. (b) Bland–Altman plot showing show the difference between ECD measurements by SM and GDOCM plotted against the mean ECD.

The average difference in ECD between the two modalities was 3.7%, and their Pearson correlation had an $R^{2}$ of 0.91 (p<0.001), as shown in Fig. 15(a).

The difference in mean ECD between the two modalities was $99\;{\text{cells}/\mathrm{mm}}^{2}$, and Bland–Altman analysis revealed that 95% of the differences in ECD were within $\pm 268\;{\text{cells}/\mathrm{mm}}^{2}$ of the mean difference, as shown in Fig. 15(b). The Bland–Altman 95% confidence interval includes zero.

The entire pipeline for automated cell counting from GDOCM images, segmentation, postprocessing, and counting, takes an average of 2 s per cornea on a common laptop (a MacBook Pro with a 3.1 GHz Intel Core i7 processor and 8 GB of memory). The memory footprint of the trained network is about 122 MB. By comparison, a trained technician takes on average 20 to 30 min to perform SM evaluation.

## 4 Discussion

For the first time, corneal tissue stored in viewing chambers was imaged with noncontact modality using GDOCM, a 3D numerical flattening procedure was developed to produce an artifact-free *en face* view of the endothelium, and machine learning was applied to GDOCM images to automatically count endothelial cells.

The results presented in Fig. 10 show an excellent agreement with the manual annotation, even with the small training set available for this study; no statistically significant difference in mean ECD was found, as indicated by the fact that the 95% confidence interval of Fig. 11(b) included zero. Image quality affected the segmentation of cornea 2, as can be seen from the higher standard deviation in ECD (C2, L3 in Fig. 11). The accuracy of the segmentation can be improved using a more powerful classification model, such as one based on U-Net,^47^ and a more sophisticated postprocessing approach, such as morphology analysis.^48^

The combination of GDOCM imaging and CNN automated cell counting was shown to produce results that are not statistically significantly different from the current practice of semiautomated counting from SM images, as indicated by the fact that the 95% confidence interval of Fig. 15(b) included zero.

Figure 9 shows that the network can identify cells not identified by the manual annotator. While this may suggest super-human performance (i.e., better than most humans at this task), which is reported in several successful machine learning models,^49^^–^^51^ additional manual examination of those cells indicated the need for revisiting the ground truth to ensure a more accurate annotation. This review of the ground truth should be performed blindly (i.e., without having access to the automated cell count results) to avoid introducing bias during training.

Current practices are for eye bank technicians to manually select 50 to 100 cells within a much larger analysis area of $0.12\;{\mathrm{mm}}^{2}$. The cells within that area but not included in the manual selection do not contribute to the ECD estimation; thus, the effective field of view used by SM is even smaller than the $0.12\;{\mathrm{mm}}^{2}$ analysis area. Additionally, manual selection of cells for ECD estimation from SM images also suffers from selection bias (i.e., the operator usually selects the cells that look the healthiest) and interoperator variability, with the consequence that results are not reproducible between different eye banks.^7^ The proposed approach using GDOCM and machine learning has the potential to count ∼1500 to 3000 cells in $1\;{\mathrm{mm}}^{2}$ (15 to 60× improvement over the current practice, which consists of manually counting 50 to 100 cells) and eliminate selection bias. Interoperator variability derives from the imaging component only, since the automated cell counting produces the same result given the same image, and is expected to be around 2%.^32^

In this preliminary study, the same corneal tissue was imaged with two modalities (SM and GDOCM), in the same central region of the cornea, but the cell counting comparison could not be performed on the exact same cells. For this reason, a paired t-test was not appropriate in this case, since our purpose was to compare two methods (GDOCM and SM), and, while SM is considered the gold standard, it does not constitute the absolute gold truth. The count from SM is impacted by several factors, including the tissue conditions, SM image quality, and which cells are selected by the operator. We believe that the variability in cell counts obtained with the two methods is consistent with the natural variability of endothelial cells within the cornea, given that the two methods imaged cells in the same region at the center of the cornea, but not the exact same cells. Future validation will be conducted imaging the same cells with the two modalities and extending the proposed CNN for automated counting of endothelial cells from SM images. Sensitivity to image quality variations will also need to be assessed on a larger dataset; further, methods to deal with images of reduced image quality can be implemented.^24^

For future work, we plan to improve the classification model by combining U-Net with the generative adversarial networks approach.^52^ A sliding window approach can also increase the performance of the classification without requiring a larger dataset.^22^ Postprocessing can be improved by introducing morphological analysis to automatically detect undersegmented cells.

## 5 Conclusions

In this study, noncontact imaging of corneal tissue through the viewing chamber was demonstrated with Gabor-domain optical coherence tomography (GDOCM) over a larger field-of-view than SM. A 3D image processing algorithm was developed to segment the endothelium and correct the corneal curvature, resulting in artifact-free images of the flattened endothelium. The combination of GDOCM and machine learning offers unbiased estimation compared to the current procedural standard of manual cell selection on SM images.

The proposed approach combining GDOCM imaging and machine learning has the potential to increase accuracy and robustness of ECD estimation by counting all the cells visible in the ${1\text{-}\mathrm{mm}}^{2}$ field of view, thereby eliminating selection bias, and to reduce interoperator variability to ∼2%. In the future, this could lead to more robust analysis of corneal tissue health prior to transplantation, and, combined with *in vivo* imaging, assist clinicians in the assessment, and treatment of corneal disease.
